# Foreign Medical Literature

**Published:** 1818-10-01

**Authors:** 


### THIRD DEPARTMENT.

### Foreign Medical Literature.

### I.

### *Practical Observations on Scirrhus of the Stomach.*

[ From the French of Bayle and Cayol. ]

I. *Etiology.* THERE are few diseases of more frequent occurrence than Scirrhus of the Stomach. It seems to depend on the same *constitutional* disposition as cancerous diseases of other parts; but its exciting causes are more numerous and varied. The principal of these are, profound and long continued mental anxiety; excess in vinous and spirituous liquors, particularly if taken in the morning on an empty stomach; blows on the epigastrium —compression on this part; suppression of an habitual discharge, or a cutaneous eruption—in fine, whatever can determine an irritation of any kind on the organ of digestion. This disease is rarely developed before the age of 25, most commonly between the 36th and 50th years. Its effects are at first local; but at a certain stage, it deranges the process of nutrition, and all the other functions of the body.

II. *Symptomatology.* The complaint generally announces itself by a sense of uneasiness, sometimes a pleasant sensation in the epigastric region, especially when the stomach is empty. To these nascent symptoms succeed, at the end of some months, or even years, an almost constant sense of tightness; a dull and deep-seated pain in the same region, more particularly after eating. A great quantity of gas, sometimes inodorous, sometimes fœtid, is extricated in the stomach, and throughout the whole intestinal canal. The epigastric pain becomes stronger and more constant, and radiates, as it were, sometimes towards the œsophagus, the hypochondria, and the abdomen. The patient complains, besides, of a sort of anxiety, or even a real pain in the vertebral column, about the last dorsal vertebra, or in the lumbar region. He begins to vomit, from time to time, a colourless matter, aqueous or filmy, bitter or insipid, especially in the morning before breakfast. As the disease advances, he throws up his food occasionally, after eating, at first unaltered, but afterwards of a brownish colour, as though it had been macerating in infusions of tobacco, coffee, or chocolate. These symptoms, after being reiterated at longer or shorter intervals, sometimes disappear, to be again renewed at the end of an indefinite period, varying from a few weeks to six or more months. At length they become habitual, and daily acquire a greater degree of intensity; and now the sensibility of the stomach, perverted in a thousand ways, gives rise to phenomena the most singular. Aliments that used formerly to be digested with ease, now provoke nausea and vomiting; whilst other alimentary substances, apparently of the most difficult solution, occasion no accident of this kind. The man who delighted in wine will feel the utmost repugnance to this beverage But a phenomenon still more extraordinary and inexplicable is the *choice* which the stomach seems capable of making between different substances therein introduced, and the faculty it possesses of rejecting one species and retaining another, notwithstanding the state of commixture in which they may exist. We frequently see a patient vomit up shortly after a meal, the aliment he swallowed the preceding day, or even many days previously, without rejecting a particle of his last repast!

About this period, sometimes later, new derangements of the digestive functions begin to develope themselves, such as obstinate hiccup; colicky pains, analogous to those from flatulence or spasm, almost always accompanied by constipation of the bowels. On examining the abdomen, we shall generally discover in the epigastric region, a hard tumour, more or less voluminous, which appears globular or irregular; mobile, or adherent to the neighbouring parts; indolent, or somewhat sensible on pressure. At different times, this tumour may present different characters, in respect to sensibility and position; it may even sink into one or other of the hypochondria, and elude the touch, in consequence of its adhesions to contiguous viscera. It is in this way that people have vainly flattered themselves that they had *dispersed* a scirrhous tumour, when only an accidental change of position had taken place. If the scirrhus be seated anteriorly to the trunk of the aorta or cœliac artery, its communicated pulsations may cause it to be mistaken for aneurism. Another source of error which ought always to be kept in mind by the practitioner is this—from irregular and spasmodic contractions of a portion of intestine, particularly of the colon, a globular distension of gut, from pent up flatus, will often simulate, and be mistaken for, a scirrhous tumour, when the abdomen is manually examined. These windy tumours too, very generally produce an insupportable sense of uneasiness; great pain for the moment; jactitation, and want of sleep, which tend to increase the deception.

Such are the symptoms which characterize the *first period* of the malady—that is to say, before the process of nutrition has suffered any material interruption. The complexion is not yet sensibly altered—the pulse is merely a little concentrated, and smaller, during the moment of pain; the appetite keeps up in the intervals of vomiting. Sometimes, however, the two periods or stages of the complaint are blended; and from the very commencement of disordered function in the stomach, emaciation takes place, with œdema round the ankles, yellowish tint of the skin, prostration of strength, &c.

As the *second* stage advances, the skin becomes clouded and sallow, the vomiting daily more abundant and frequent, sometimes easy, at others accompanied by most painful efforts. The ejected matters are of a sooty or chocolate colour; while the constipation, flatulence, and abdominal pains augment in proportion to the other symptoms. Even at this period, some patients will experience long intervals of ease, during which the vomiting will cease; the appetite will return; the digestion will be painless, and they will even gain flesh. But these intermissions, however protracted, afford no hope of a cure, when the symptoms of scirrhus before detected have once clearly manifested themselves. Sooner or later the disease resumes its course, sometimes with accelerated rapidity, especially if the patient, under the illusion of false hope, abandons the rigid regimen previously pursued. As the emaciation increases, the features sharpen; the eyes become hollow; the epigastric tumour is more distinct from the wasting of the body. In some rare cases, where adhesions had taken place between the scirrhus and abdominal parietes, and where the tumour was soft to the feel, imprudent surgeons have plunged into it their bistoury, and penetrated the stomach instead of an abscess!

The slightest repast now causes extreme uneasiness, or even insupportable sufferings in the epigastric region, which are not relieved but by vomiting. The black matters ejected frequently contain clots of blood. The pain occasioned by flatulence, and the want of sleep are most harrassing; the pulse becomes accelerated, but there is rarely any hectic fever, where no complication exists. Finally, the patient, worn out with sufferings, which endure sometimes for several years, dies almost a skeleton.

III. *Modifications.* Considerable modifications in the symptoms result from the seat of the scirrhus being in the pyloris, cardia, or some other part of the stomach.

IV. *Pylorus.* When this is the exclusive seat of the disease, the vomitings only take place after a certain lapse of time from the period of eating. The gastric secretions and the ingesta not finding a free egress through the pyloric orifice, accumulate in the stomach, and sometimes distend this viscus extremely, before vomiting is determined, which sometimes requires a day or two, and is then copious. The site of the tumour in the *right* epigastric region, between the false ribs and the umbilicus, will aid in recognizing pyloric scirrhus.

V. *Cardia.* If the scirrhus be situated at the cardiac extremity of the stomach, no epigastric tumour will be distinguishable. The pain will be chiefly felt at the upper part of the pit of the stomach; the deglutition will be occasionally difficult; the vomitings almost immediately supervene on eating; the food sometimes returns into the mouth without reaching the stomach; some patients disgorge great quantities of viscid mucous like saliva. Still there is considerable uncertainty in pronouncing the exact seat of scirrhus.

VI. *In other Parts of the Stomach.* Here the vomitings are, generally speaking, but little observed, except at the very beginning of the disease, and when adhesions have subsequently formed between the morbid and the neighbouring parts. But constipation, flatulence, and the other symptoms of gastric scirrhus are present here as in the other species.

But we may here again caution the practitioner against hasty or decisive opinions relative to the seat, or even the *existence* of gastric scirrhus, since in opening patients who have died of general marasmus, without evincing a single symptom of vomiting, pain, nor even dyspepsia, we have repeatedly found scirrhi and even open cancers of great extent in the stomach. Among the indigent classes of society, sensibility is so blunted by sufferings and privations of every kind, that these *occult organic lesions* frequently conduct their victims in silent emaciation to the tomb, without materially troubling the *functions* of the viscera affected.

Another source of error is the *cough* which sometimes accompanies gastric scirrhus, attended with more or less expectoration. It occasionally comes on in such fits as to provoke vomiting. In such circumstances, if the symptoms of gastric scirrhus happen to be faintly or irregularly marked, the disease is put down as pulmonary phthisis, and dissection shews the lungs sound, and the stomach disorganized by cancer!

All that Physiologists have said respecting the remarkable *mental despondency* which accompanies organic lesions of the viscera seated in the hypochondriac regions, has appeared to us to be very vague, uncertain, and often erroneous. The said despondency more frequently attaches to the *neuroses,* or hypochondriacal affections, than to the *organic* derangements of those parts. Among the numerous patients which we have treated for gastric scirrhus, we have not found a greater proportion affected with mental depression than in other serious internal maladies, except where the *pain* was excessive, or the *dyspepsia* obstinate; for it is with this *last* phenomenon that lowness of spirits so frequently associates.

VII. *Pathology.* Dissection shews, in gastric scirrhus, the same morbid appearances that present themselves in scirrhus of the œsophagus, pharynx or rectum. The stomach is generally filled with a blackish sooty liquid, like coffee or chocolate infusion, united with alimentary matters. This is not a fluid secreted, as some suppose, from the ulcerated parts; because it is frequently found where there is only scirrhus, and not always where there is actually cancer. Where the disease is seated at or near the pylorus, the stomach is enlarged and dilated—otherwise it is contracted. The extent of the diseased portion varies from the size of a sixpence to that of the whole viscus. It is usually, however, about the size of the palm of the hand. The *internal* surface of the scirrhous or cancerous part is generally prominent and uneven, and it is here that the ulceration always begins.

III. *Diagnosis.* 1. When *chronic inflammation* of the stomach has continued a long time, and reduced the patient to a state of emaciation, it is sometimes extremely difficult, if not impossible, to distinguish the disease from scirrhus; especially if it be unaccompanied by fever, and attended with vomiting of dark-coloured fluids, as we have often observed it to be. In such cases of embarrassment, we may presume, that the disease is chronic inflammation, and *not scirrhus,* if it commenced all at once with vomiting; if antiphlogistic remedies procured relief of the symptoms; if the subject be under twenty-one years of age; if the vomitings are so very frequent, that the stomach appears incapable of bearing the contact of aliment; if, at the end of several months of almost continual vomitings, with rumination or regurgitation of the food, no tumour can be distinguished in the epigastric region; finally, if the patient, although considerably emaciated, has not that peculiar yellowish tint which characterizes the cancerous cachexy.

Fortunately an accurate *diagnosis* between these two diseases is not of vital consequence, as the treatment for chronic phlegmasia will agree with scirrhus; but in the *former* it may effect a cure, while in the *latter,* it can be only paliative. So far the *diagnosis* is serviceable on account of the *prognosis.*

2. *Spasmodic vomiting,* a disease essentially nervous, and often resulting from moral causes, becomes occasionally *chronic,* with emaciation, discharges of dark-coloured fluids, and even death. These *last* cases, though rare, may be mistaken for gastric scirrhus, and the diagnosis is not very easy. In general, spasmodic vomiting admits of cure, and when it terminates mortally, no trace of scirrhous disorganization is to be seen. It is only by a careful survey of the causes, the symptoms, and the effects of different remedies, that we can distinguish chronic cases from the dreadful disease under consideration.

3. A *perforation* of the stomach may, and does result from a chronic phlegmasia of this organ. But the adhesions which take place, during this slow inflammation, prevent any effusion into the general cavity of the abdomen; yet still an ulcer exists, with loss of substance, which causes epigastric pains, vomitings, progressive emaciation; in fine, the greater number of symptoms which accompany gastric scirrhus. It requires great attention to the history of the complaint to distinguish it from the disease under review. It generally commences with inflammatory symptoms, and is owing to an external cause.

4. *Biliary concretions*, giving origin to vomitings, colic, constipation, emaciation, &c. with an epigastric tumour in the pyloric region, have been mistaken for scirrhus; but here the matters vomited are hardly ever black. Besides the accompanying jaundice, the appearance of the stools, and the nature of the pains leave little room for deception, where common attention is paid to the complaint.

5. Tumours, not scirrhus, situated in the vicinity of the stomach, may give rise to symptoms resembling those of scirrhus of that organ, and these can only be ascertained and distinguished *post mortem*.

IX. *Treatment*. The first object is to find out the occasional cause, and remove it if possible. Thus, if the patient be a mechanic, whose trade keeps an habitual pressure on the epigastric region, he is to be advised to change his profession. If a drunkard, he is to be warned against inebriating drink. If, previously to the gastric affection, he was subject to a cutaneous eruption, hæmorrhoids, or habitual discharges of blood, every means must be put in force to restore these outlets. But of all occasional causes of gastric scirrhus, the most frequent of occurrence, and the most difficult of removal are the sombre moral affections of the mind.

When the disease is developed in a female, about the period of catamenial cessation, we may hope to check the rapidity of its progress by bleedings and derivatives.

Of the general remedies which have been proposed in mitigation of gastric scirrhus, the extracts of cicuta and hyoscyamus have appeared to us the most useful. But while we study the action of different remedies, we must attentively watch the march of the disease, in order not to counteract the conservative efforts of Nature. The symptoms of gastric scirrhus, like those of most other organic diseases, experience sometimes a sort of intermission; thus we have known persons who, to all appearance, were in perfect health, although a large scirrhus had been established for many years, and which at length terminated fatally. It is in spring and autumn particularly, that the relapses or exacerbations of the disease take place, and, therefore, we are to vary our treatment according to these revolutions.

Unfortunately no means have yet been found out to arrest the *march* of scirrhus in the stomach; and all we can do is to mitigate the *symptoms.*

*Flatulence.* When this appears to be the principal cause of pain, opium, hyoscyamus, ether, peppermint, and other antispasmodics are useful. Aromatic and anodyne fomentations to the epigastrium have dissipated a painful distention of the stomach when all other means had failed. Ice drinks, and the application of ice to the pit of the stomach have also given ease in distressing cases. The irritability of the stomach may often be lessened by bland diet of easy digestion; by infusions of poppy heads, mint, orange flowers, &c. When there appears a superabundance of mucous secretion in the stomach, which deranges the digestion by commixture with the aliments, magnesia, the absorbent powders, the extracts of bitter plants, &c. will prove serviceable.

*Constipation,* when it occasions a painful abdominal distention by preventing the exit of flatus *per anum,* is best obviated by mild glysters, rather than purgative or laxative medicines exhibited internally.

*Regimen.* It is principally by studying the idiosyncracy of the individual, and adapting the lightest and most easily digestible food to the particular case, that the ravages of this terrible disease are to be suspended, or at least protracted. It is impossible to lay down any general rule here; but perhaps there is no alimentary substance which so universally agrees with gastric scirrhus, as milk. To prevent its curdling in the stomach, a small proportion of lime water will be found a valuable addition. A diet of this kind, with pure or distilled water, has often signally ameliorated the state of patients labouring under this cruel disease, even where emaciation had advanced to an alarming degree.

### II.

### *On the Dangers of Dissection.*

By M. Percy.

The accidents to which the Anatomist is liable in the prosecution of his studies may be divided into two classes: those resulting from the putrid gases extricated from the dead animal matters, and acting on the system generally; and those from inoculation of a septic principle, in wounds.

1. Cullen had long ago observed, that anatomical students enjoyed excellent health, in general, notwithstanding their being so much among the exhalations from dead bodies. Professor Bosquillon in one season dissected nearly 600 bodies, of all ages, sexes, and conditions; and states, that of five hundred pupils who passed from six to eight hours daily in dissecting, three only contracted disease from thence, and but one died. It is very different with them in their pathological investigations. Out of one hundred pupils employed in hospital service, sixty were seized with hospital fever. But we are not to infer from these premises, that no danger attends the dissection of bodies. A well-known and melancholy example will prove the contrary. Dr. Chambon, in order to demonstrate the liver and its appendices, laid open the abdomen of a subject considerably advanced in decomposition. A horrible gas immediately issued forth, and nearly overwhelmed the demonstrator and four others. One of these, M. Corion, fainted away, was carried home, and died in sixty hours! The celebrated Fourcroy, who was also present, would, it is supposed, have shared the same fate, had it not been for an exanthematous eruption which was strongly out on him at the time. Messrs. Laguerenne and Dufresnoy remained long ill, and the latter was never restored to health afterwards. The Professor escaped best, although he proceeded on with the dissection. In the night he had a fever, which ended in a copious perspiration towards morning, and left him free from complaint.

At Dijon, in 1773, the accidental opening of a coffin in which a body had been six weeks buried, affected 114 people dangerously, and of whom 18 died. Many individuals have perished from the escape of animal gases of this kind; instance the grave-digger of Montmorency. We shall not recount what passed during the evacuation of the Cemetery of the Innocents, where, among others, Thouret contracted a fever that nearly terminated his existence.

But, fortunately, these instances are rare, and we see thousands of Anatomists enjoy good health among corses in all states of putrefaction. Who had dissected more, or prosecuted pathological anatomy more, than our venerable colleagues Tenon and Portal, who are blessed with a green old age? Look also at Walter, Mascagni, Scarpa, Sœmmering, Pelletan, Deschamps, Laumonier, Chaussier, Boyer, &c. who have spent nearly half their lives among dead bodies, and yet promise a happy longevity.

It is said that Claude Perrault fell a sacrifice to the dissection of a putrid camel; and that Taren shared the same fate from demonstrating a human body. We may add, that the memorable Bichat would now have been living, had he not given himself up too much to dissection and macerations of morbid parts. Indeed, we see students daily destroyed by too enthusiastic a pursuit of anatomical studies.

2. But it is from inoculation of dead matters that the great danger is to be apprehended. Dr. Chambon relates, that having pricked his middle finger with the sphænoid bone of a skull that had been long in maceration, he was soon afterwards seized with the most intolerable pain, and inflammatory swelling of the fingers and hand, and with a number of acute and uneasy sensations, which he compares to those of the most violent gout. He experienced, in 1810, a similar train of accidents, from dissecting a body while there was a slight excoriation of the middle finger. Constitutional symptoms of great violence supervened, with disturbance of the mental faculties, great irregularity of the pulse, and extreme debility.

It has been long observed, that one of the most striking phenomena attending the accession of plague and other pestilential fevers, was a peculiar mental despondency, and disturbance of the sensorial functions. The very same takes place in the inoculation of septic poison in dissection, as was almost fatally exemplified in the person of the illustrious Corvisart, in 1786. This distinguished ornament of the profession pricked his finger while inspecting a dead body. Presently the whole arm swelled to an enormous size. Desault was obliged to make repeated and deep incisions in the tumefied parts, which Corvisart bore with considerable firmness, although he had experienced the peculiar mental despondency appertaining to the disease, even to *despair*.—"Circonstance qui affligea plus vivement les temoins assidus de sa triste situation, que tous les autres ravages qu'avait produits le virus inoculé."—Finally, however, the skill of Desault (whose friendship for the patient did not arrest the salutary course of the knife, or prevent him from acting the determined surgeon), triumphed over the effects of the poison, and restored to health our beloved colleague.

In these accidents, the train of symptoms is not always equally *painful* and dangerous. I knew a student, who, having cut himself while tracing the nerves on a subject which he had kept for some weeks, died in three days, in a state of the greatest debility, but without experiencing any pain, although gangrene occupied the whole arm. In other instances death has taken place at a much earlier period. It was probably in this way that Professor Leclerc perished in thirty-six hours, after feeling the pulse of a patient ill with malignant fever. His finger was excoriated, and the sick man's arm was covered with perspiration. Perhaps, however, his death might be owing, as in the case of Chorion before mentioned, to the *pulmonary* absorption of a deleterious gas from the diseased body.

M. Chambon states, on the authority of several historians, and on the testimony of the President De Thou, that the Peruvians, animated with just vengeance against the Spaniards, dipped their arrows in the sanies flowing from the putrifying bodies of their unfortunate comrades, in order to render the wounds of their oppressors more certainly and more speedily fatal.

M. Huzard observes, that no carcases putrify so quickly, and emit so much dangerous exhalation, as those of herbivorous animals, as the horse, the ox, &c. He has seen numerous fatal instances of this poison among the veterinary students, when they happen to wound themselves in dissecting these animals, even within three or four days after death.

*Treatment*. We may here observe, with the celebrated Fabricius Hildanus, that it is not the virulent matter of poison, such as it appears to the eye, which is so quickly absorbed, and proves so rapidly fatal. It must be some subtle principle contained in it, and which eludes the sight. This view of the subject leads to the surest mode of arresting the evil, viz. either by removing the part, or destroying its organization, so that the process of absorption may be prevented. It is the cautery, either actual or potential, that the best anatomists of the present day rely on. As the wounds of dissection are generally small, a red hot needle will usually be sufficient. But, as these wounds are sometimes made by needles, or the points of hooks, it is not so easy to apply the cautery to their bottoms. Here we may take a lesson from mechanics, who, when they prick their fingers or hands with pointed instruments, immediately throw a little oil on a piece of burning coal or charcoal, and hold the wounded part over the smoke, which cauterizes the wound completely.

For several years past, the precept and example of Professor Chaussier have been adopted in the anatomical schools of Paris; viz. Each student keeps constantly in his pocket a small phial of liquid butter of antimony (muriate of antimony), and whenever he wounds himself in dissecting, plunges the point of a little wooden pencil into the caustic, and instantly cauterizes the puncture or wound. It is dangerous to wait till the actual cautery can be got ready. We ourselves always recommend the *strong nitric acid*, where the wound is tortuous, or made with a sharp-pointed instrument, as this liquid immediately penetrates through every part of the puncture, and completely disorganizes its parietes, and renders them incapable of taking up any part of the septic principle.

When unfortunately the poison has taken effect, we know of no *specific* means of checking its progress, and are obliged to combat the symptoms on general principles, for which no rule can be laid down. It is to be hoped, however, that the prudent precautions of the Parisian schools will be imitated in those of Great Britain, where we know, from painful personal experience, that dissection wounds are too much despised and neglected; or at best but washed clean, and touched with nitrate of silver, which is by no means an effectual cautery.

